# Relative efficiency of psychiatric clinics in treating cases without coercion and achieving symptom reduction

**DOI:** 10.1192/j.eurpsy.2025.10034

**Published:** 2025-05-27

**Authors:** Cornelius Müller, Tiziana Ziltener, Julian Moeller, Roselind Lieb, Undine Lang, Christian Huber

**Affiliations:** 1 University Psychiatric Clinics (UPK) Basel, Clinic for Adults, University of Basel, Basel, Switzerland; 2 Faculty of Psychology, Division of Clinical Psychology and Epidemiology, University of Basel, Basel, Switzerland

**Keywords:** adolescent psychiatry, coercion, hospital personnel management, organizational efficiency, outcome assessment (health care)

## Abstract

**Background:**

The use of coercive measures is an increasingly debated aspect of psychiatric treatment. Considering the multitude of negative effects, patients, clinicians, and ethicists alike have called for a more cautious application of coercion. It therefore remains important to investigate which organizational characteristics have the potential to facilitate efficient coercion reduction. The same holds true for the efficient reduction of symptom severity during inpatient treatment.

**Methods:**

The current study compared 22 Swiss psychiatric clinics treating 45,095 cases regarding their relative efficiency in treating cases without coercion given their staff resources. To this end, we applied a Data Envelopment Analysis to clinical routine data. We focused specifically on inefficiencies attributable to management factors independent of the clinics’ total staff numbers. We further compared the clinics’ relative efficiencies regarding changes of self-reports and third-person reports of symptom severity during inpatient stays.

**Results:**

Efficiency scores suggest that on average, the clinics could improve the percentage of cases treated without coercion by 9% and the changes of symptom severity by 34% (for third-person ratings) or 18% (for self-reports) while keeping staff numbers constant. An analysis of specific coercion types revealed that the potential for efficiency improvements via management was highest for movement restrictions. We found no effect of clinic size on efficiency scores regarding any of the outcome measures.

**Conclusions:**

Our results underline the importance of management factors beyond staff resources (e.g., staff trainings or changes in ward structure and treatment concepts) for the efficient reduction of coercion and psychiatric symptoms during inpatient stays.

## Introduction

The use of coercive measures, i.e., clinical interventions against a person’s will [1] is an increasingly debated aspect of psychiatric treatment. In qualitative studies, patients have addressed that their autonomy is often restricted to an unnecessary extent [2]. In agreement with this, patient representatives, clinicians, ethnicians, and other stakeholders have called for a more cautious application of coercive measures [3–5].The World Health Organization’s (WHO) Convention on the Rights of Persons with Disabilities laid the ethical and legal ground for coercion reduction [6]. Later, the WHO Quality Rights Initiative declared freedom from coercion a central aim for the progression towards human rights-based mental health treatment [7]. Past studies reported multiple adverse effects of coercion. The application of coercive measures elicits a wide range of negative emotions in patients and staff alike [2, 8]. As a direct consequence of coercive measures, patients can experience physical injury [9] and symptoms of posttraumatic stress [10]. Further, the use of coercion is associated with increased length of inpatient stay [11], worse therapeutic relationships [12], higher treatment-seeking threshold [13] and stronger stigmatization of patients [14]. At the same time, the effectiveness of coercive measures to reach the goals justifying their application, i.e., to prevent self-harm or danger to others, is contested by empirical findings [15, 16]. While patient characteristics like the type and severity of mental disorders appear to be strong predictors for the application of coercion during inpatient stay [17], there is considerable variability in the use of coercive measures between psychiatric clinics [18–20]. This highlights the importance of identifying organizational characteristics that contribute to the use of coercion.

Several authors suggested an increase of staff numbers as a possible means to reduce coercion [21, 22]. This is in line with suggestions from the patient perspective [23]. To the contrary, Patel et al. argued that a mere increase of available resources per person does not effectively counter large-scale mental health issues [5]. Empirical findings on the association between staff numbers and the use of coercive measures are inconsistent. While some studies support the idea that increases in staff resources could reduce coercion [24, 25], others did not find this effect [18, 26]. Besides an increase of staff numbers, past research provides evidence that other, management-related factors are linked to a reduction in coercion [27]. For example, de-escalation trainings educate staff members in risk assessment and provide behavioral alternatives to an early application of coercive measures when dealing with agitated or aggressive patients. Open door policies redistribute workloads away from door-monitoring towards more time spent addressing patient needs [28]. l Both de-escalation trainings and open door policies are associated with reduced coercion rates [16, 19, 29]. To transfer these findings into effective public decision-making, it is important to identify opportunities for coercion reduction among clinics on the national level. To this end, it is important to differentiate how much management factors as opposed to a mere increase of staff resources may contribute to the reduction of coercive measures.

The secondary focus of the current study was on changes of symptom severity during psychiatric inpatient treatments. Few studies exist on clinic-level variables affecting symptom reduction in psychiatry. Mahon et al. found a clinic effect on symptom changes, but their sample did not include potential explanatory variables for this effect [30]. This research gap transfers to a lack of practical recommendations. For example, a German national S3 guideline for the reduction of coercive measures exists, but no comparable guideline for the structuring of a clinic that best supports effective symptom reduction. Similar to coercion reduction, we regard it important to evaluate whether organizational changes related to clinic management could improve symptom reduction during psychiatric inpatient stays.

Past studies mostly focus on coercion and symptom severity in absolute terms. For public decision-makers operating with limited resources, it is important to improve psychiatric treatment in a cost-efficient manner. We therefore applied a Data Envelopment Analysis (DEA) to routine data collected by Swiss psychiatric clinics. DEA is a method typically used in economics to compare different organizations regarding their efficiency in converting inputs into outputs [31]. In research on psychiatry, DEA has been applied to primarily to compare clinics with respect to the staff resources needed to treat a certain number of patients [32, 33], less often to investigate clinical outcome measures [34]. In one previous study, DEA has been applied to coercion as outcome measure in a set of German hospitals [27]. To our knowledge, this is the first study applying DEA to outcome measures regarding coercion and changes of symptom severity in Switzerland.

### Research questions

The current study aimed to compare the relative efficiencies of Swiss psychiatric clinics regarding the following research questions: How large is the potential of Swiss psychiatric clinics to reduce

- coercive measures
- symptom severity

with management-driven changes while holding staff numbers constant?

## Methods

### Data sources

We retrieved data on Swiss psychiatric clinics from two publicly available datasets. To rule out possible effects of the COVID-19 pandemic on coercion, we focused on the year 2019. We retrieved the percentage of cases treated with coercion, measures regarding specific coercion types,as well as average values of self-reported and third-person reported symptom severity per clinic from the yearly report by the Swiss National Association for Quality Development in Hospitals and Clinics (ANQ; [35]). We retrieved staff numbers from the Key Figures for Swiss Hospitals published by the Federal Office of Public Health [36]. Since all data were collected as part of clinical routine procedures and made publicly available by the above-mentioned institutions, no additional consent form was needed.

Out of 44 clinics providing basic and acute psychiatric inpatient care in Switzerland, we included only those applying coercive measures, leaving 37 clinics for the analysis., Six clinics were excluded as they did not report numbers on coercive measures due to issues with data transcription (technical issues with the transcription of the data or data only available for specific wards). For some clinics providing care in different medical sectors, staff numbers were not available specifically for the psychiatry sector. For this reason, another nine clinics were excluded. For the analysis of self-reported symptom severity, one further clinic was excluded due to missing values. For the analysis of third-person reported symptom severity, a different clinic was excluded as an outlier. Thus, 22 clinics were eligible for the analysis of coercive measures and changes in symptom severity (see Analysis for final sample sizes after outlier exclusion).

### Measures

The Federal Office of Public Health reports staff numbers in full-time equivalents (FTEs), categorized into nursing staff, physicians, and other staff (e.g., medical staff, psychotherapists, physiotherapists, occupational therapists, nutritionists).

In a standardized procedure, Swiss psychiatric clinics report all incidents of formal coercion including seclusion, restraint, and treatment without consent. The ANQ report summarizes these measures as percentage of cases treated with at least one coercive measure. We included measures of the following specific types of coercion as additional outcomes: seclusion, fixation, coercive medication, and movement restrictions to bed or chair. Incidents of holding were not included since only a small fraction of the clinics had applied this. Including only cases experiencing at least one of the respective coercive measures, the cumulative duration of seclusions and fixations was quantified as the average *time (in h) x frequency*, whereas coercive medications and movement restrictions were reported as average incidents per case.

Third-person reports of symptom severity were assessed with the Health of the Nation Outcome Scales (HoNOS; [37]). TheHoNOS allows clinicians to rate patients in terms of clinically relevant behavior, impairments, symptoms, and social functioning. It comprises 12 items with five-point rating scales. Total scores range from 0 to 48. HoNOS has demonstrated moderate internal consistency, as well as adequate test–retest and interrater reliability [38]. HoNOS ratings are routinely carried out by the physician responsible for a patient once at patient admission and once at discharge. Training courses for HoNOS ratings are offered by the ANQ, but not obligatory for physicians applying it in daily practice. We investigated HoNOS difference scores between patientadmission and discharge. Across all included clinics, mean response rates including non-influenceable dropouts (e.g., due to short durations of stay) were 97.5%, ranging from 78.2 to 100%.

Self-reports of symptom severity were assessed with the Brief Symptom Checklist (BSCL; Franke, 2017). The BSCL measures patients’ subjective physical and mental symptom severity on a five-point rating scale. Total scores range from 0 to 212. The BSCL has demonstrated moderate to high internal consistency and moderate to good test–retest reliability [39]. We investigated BSCL difference scores between admission and discharge. Across all included clinics, mean response rates including non-influenceable dropouts (e.g. due to short durations of stay or too severe impairment) were 83.5%, ranging from 59.6 to 100%.

Due to systematic differences regarding the frequencies of specific diagnoses between clinics, the ANQ reports both HoNOS and BSCL differences adjusted for case mix. The ANQ does not apply this adjustment procedure to the numbers on coercion. In response to a request, the ANQ informed us that the adjustment was not carried out because pre-examinations showed that adjusted values would only differ marginally from unadjusted ones.

As all data sources are readily available in the public domain, no ethics committee vote was needed for the current study.

### Analysis

We calculated Spearman’s rank correlation coefficients to investigate the associations between the different coercion types, as well as between the relative measures of coercion and absolute coercion numbers. We applied Holm’s correction for multiple comparisons to the p-values of the respective correlation coefficients.

#### Data envelopment analysis – Background

DEA is a nonparametric method that allows comparisons of so-called decision-making units (DMUs) based on their efficiency in converting input variables into outputs. To this end, DEA computes a convex efficiency frontier. DMUs located on the frontier have a technical efficiency (TE) score of 1, or 100%. DMUs with efficiency scores below 1 are enveloped by the frontier [31]. TE is a relative measure of efficiency. While a DMU with a TE score of 1 (or 100% relative efficiency) is among the most efficient in the examined sample, it may still have potential for improvement.

We conducted DEA with a variable returns to scale (VRS) model [40]. VRS models compute a TE score that is independent of the DMU’s scale (i.e., the total number of inputs). Thus, inefficiencies detected with a VRS model are attributed to management factors.

Another important step of DEA model specification is the selection of the model’s orientation. DEA models can be computed with input orientation or output orientation. In output-oriented DEA, TE scores indicate how much outputs may be increased while holding inputs constant [31]. For example, an efficiency of 80% in output-oriented DEA would indicate that this DMU could increase its outputs by 20% while holding inputs constant.

Methodological investigations revealed that in traditional DEA, TE scores are positively biased [41, 42]. We therefore applied a bootstrap procedure for bias-corrected TE scores to increase robustness of DEA results and validity of interpretations. [43, 44].

#### Data envelopment analysis – Implementation

As we were interested in inefficiencies attributable to management factors independent of the clinics’ scale (i.e., total staff resources per 100 beds), we computed DEA models with VRS. We assume that the investigated clinics have less immediate control over staff numbers than over the respective output. Therefore, we specified all models with output orientation.

We bootstrapped three models, applying DEA to each of three different outputs. While it is possible to include separate outputs into a single DEA model, we opted for multiple models in order to obtain separate TE scores for each output variable. Each model consisted of three inputs and one output. Inputs included the FTEs for three categories of clinical staff: physicians, nursing staff, and other staff. To adjust inputs for clinic size and occupation rate, we converted all input values to FTEs per 100 occupied beds. Since DEA is designed to maximize outputs, we converted EFM scores to the percentage of cases treated *without* coercion as output variable. To obtain a more detailed picture of the clinics’ efficiency in reducing coercion, we added DEA analyses for measures of specific coercion types (see Measures subsection). Since DEA is designed to maximize outputs, these variables were reverse-coded by subtracting each value from a constant above the maximum. Total coercion numbers were not included in DEA to avoid a mismatch between relative input and absolute output measures. For the analysis regarding our secondary research question, DEA models with the same inputs included the HoNOS difference and BSCL difference as output variables.

After exclusion of statistical outliers regarding the respective outcomes, final sample sizes were: *N =* 22 for the percentage of cases treated without coercion; *N =* 22 for the HoNOS difference; *N =* 21 for the BSCL difference; *N =* 18 for reversed seclusion cumulative duration; *N* = 21 for reversed fixation cumulative duration; *N =* 20 for reversed coercive medications per case; *N =* 22 for reversed movement restrictions per case. No DEA-specific outlier clinics were found with the super-efficiency method [45]. All DEA models were computed using the rDEA package [46] in R Bootstraps were carried out with 1000 replications as suggested by the developers of bootstrapped DEA [47].

#### Truncated regression

We regressed the reciprocals of TE scores of the three different models on the number of inpatient cases in 2019 as a proxy for clinic size. Our procedure followed the suggestions by Simar and Wilson for computing left-truncated regression with TE scores [43]. Truncated regression was carried out with the truncreg package [48] in R [49].

## Results

For descriptive statistics of all input and output variables, see Table 1.

**Table 1.** Descriptive statistics

Clinic number	Physicians	Nurses	Other Staff	Total staff	Mean length of stay	Inpatient cases	HoNOS Differ-ence	BSCL Differ-ence	% of Cases treated without coercion
1	30.71	116.54	47.78	195.03	30.38	4,184	7.03	27.61	92.07
2	29.22	113.17	25.73	168.12	32.77	719	10.91	33.28	92.77
3	39.56	118.26	54.62	212.44	32.17	2,565	6.88	32.51	88.3
4	18.96	93.08	38.45	150.49	31.88	3,366	8.86	33.73	90.76
5	18.2	78.44	23.07	119.71	34.46	1,550	8.76	28.35	97.35
6	23.54	78.33	32.24	134.11	38.85	855	7.8	29.79	98.6
7	38.74	114.6	51.37	204.71	35.19	2,271	6.21	35.54	89.21
8	32.97	117.12	59.82	209.91	33.11	2,539	8.74	30.05	95.19
9	50.63	120.41	31.48	202.52	24.5	2,354	10.15	40.17	90.14
10	30.24	116.83	35.04	182.11	38.96	1,123	8.51	35.09	87.09
11	32.25	130.94	33.57	196.76	34.35	1,999	7.47	35.17	89.59
12	38.38	117.57	43.01	198.96	35.33	1,542	7.59	34.14	90.86
13	33.12	108.32	24.83	166.27	39.97	2,337	10.24	36.2	88.92
14	23.51	91.03	35.64	150.18	36.88	1,864	8.29	37.47	93.67
15	17.6	107.23	9.24	134.07	25.3	1,906	10.37	26.67	93.49
16	12.47	53.43	8.41	74.31	34.31	923	11.1	34.7	93.82
17	20.86	65.42	10.92	97.2	35.3	436	10.43	32.71	97.71
18	91.53	171.28	52.25	315.06	27.95	865	10.8	NA	94.8
19	41.45	89.37	57.49	188.31	35.9	1,325	6.33	37.53	86.42
20	45.83	155.7	52.63	254.16	34.54	5,105	1.74	30.66	91.87
21	27.43	108.51	32.63	168.57	30.41	2,758	7.99	28.61	90.61
22	29.29	92.54	30.39	152.22	26.84	2,509	8.13	29.52	95.3
Total						45,095			
Mean	33.02	107.19	35.94	176.15	33.15	2,049.77	8.38	32.83	92.21
*SD*	16.26	26.81	15.33	51.77	4.23	1,144.11	2.12	3.67	3.39
Median	30.48	110.84	34.30	175.34	34.33	1,952.50	8.40	33.28	91.97

Clinic number	Number of coercive measures	Number of isolations	Cumulative duration of isolations	Number of fixations	Cumulative duration of fixations	Number of coercive medi-cations	Coercive medi-cations per case	Number of move-ment restric-ions
1	332	286	87.55	108	31.38	114	2.11	9
2	52	51	74.79	1	77.3	25	2.04	1
3	300	239	92.68	27	26.37	134	4.95	11
4	311	248	193.62	106	48.93	133	2.19	3
5	41	36	193.76	14	90.86	32	1.47	2
6	12	12	106.03	0	0	1	1	0
7	245	180	31.65	26	14.04	106	2.28	39
8	122	112	38.75	0	0	65	1.68	3
9	232	125	73.75	29	18.9	108	2.08	69
10	145	129	41.08	0	0	67	1.21	56
11	208	174	61.06	31	11.62	37	1.19	32
12	141	122	83.68	22	23.47	23	1.43	2
13	259	214	82.29	14	15.85	121	1.67	48
14	118	115	65.41	10	69.63	0	0	0
15	124	0	0	0	0	124	1.55	0
16	57	2	65.83	10	49.32	44	3.27	11
17	10	9	250.89	7	185.61	0	0	1
18	45	14	250.62	0	0	31	1.77	1
19	180	142	45.8	19	16.06	57	2.4	30
20	415	346	29.04	9	31.32	239	13.39	27
21	259	211	81.47	3	57.35	91	2.66	19
22	118	111	12.73	0	0	61	1.67	0
Total	3726	2878		436		1613		364
Mean	169.36	130.82	89.2	19.82	34.91	73.32	2.36	16.55
*SD*	113.54	99.13	70.48	30.11	43.23	58.07	2.67	20.78
Median	143	123.50	74.27	10	21.18	63	1.73	6

Clinic number	Movement restrict-ions per case	Number of holding events	Cumulative duration of holding
1	11.56	4	0.18
2	1	0	0
3	1.55	0	0
4	5.67	0	0
5	1.5	0	0
6	0	0	0
7	6.1	18	1.48
8	10	0	0
9	6.86	23	1
10	1.05	5	0.28
11	6.19	0	0
12	1	0	0
13	8.65	0	0
14	0	0	0
15	0	0	0
16	8.45	0	0
17	1	0	0
18	1	0	0
19	15.17	0	0
20	7.22	0	0
21	1.11	0	0
22	0	0	0
Total		50	
Mean	4.32	2.27	0.13
*SD*	4.46	6.1	0.37
Median	1.52	0	0

*Note:* Nurses = full-time equivalent (FTE) of nurses per 100 occupied beds; physicians = FTE of physicians per 100 occupied beds; other staff = FTE of medical-technical and medical-therapeutic staff per 100 occupied beds. Total staff = FTE of all staff types combined per 100 occupied beds. Cumulative durations were computed as frequency x time (in hours) on the case level. For measures of specific coercion types, only cases in which the respective type of coercion was applied at least once were included.

Abbreviation: NA, not available.

**Figure 1.**
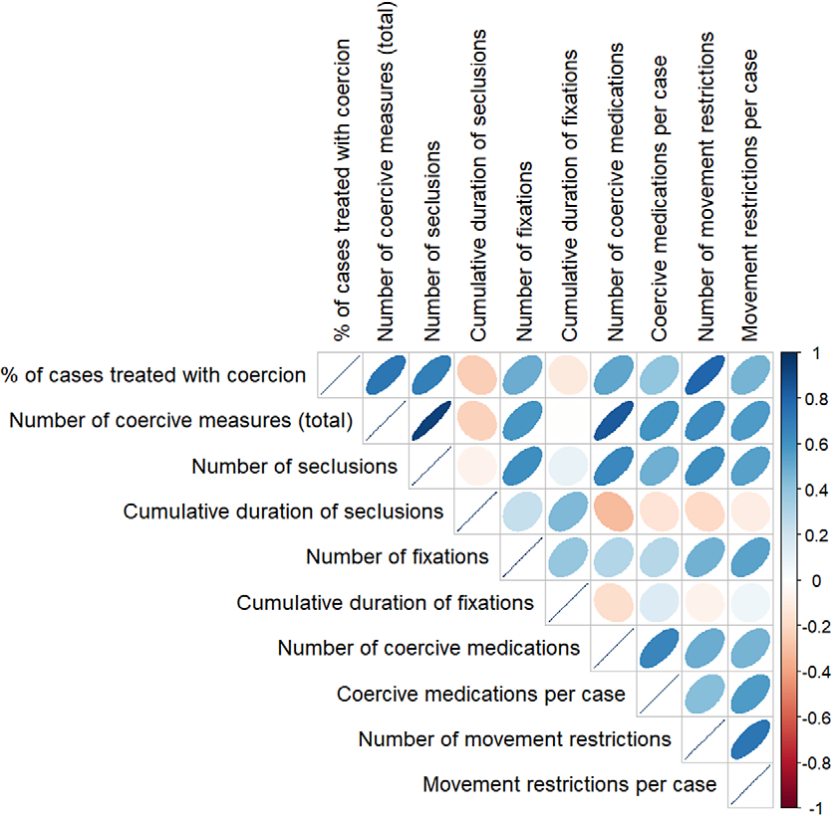
Spearman’s rank correlations between different measures of coercion. *Note:* Colors indicate Spearman’s Rank Correlation as indicated in the bar on the right. Size of ellipses visualize strength of the association.

Figure 1 shows Spearman’s rank correlation coefficients between different measures of coercion. After Holm’s correction, we found significant correlations of the percentage of cases treated with coercion with the total number of coercive measures (*r*(20) = .72; *p* < .01), the number of seclusions (*r*(20) = .69.; *p* = .016), and the number of movement restrictions (*r*(20) = .79; *p* < .001). Further, the total number of coercive measures correlated significantly with the number of seclusions (*r*(20) = .92; *p* < .001), and the number of coercive medications (*r*(20) = .83.; *p* < .001). The number of seclusions correlated significantly with the number of coercive medications (*r*(20) = .64.; *p* < .047). The number of coercive medications correlated significantly with the number of coercive medications per case (*r*(20) = .65; *p* = .04) and the number of movement restrictions with the number of movement restrictions per case (*r*(20) = .73; *p* < .01).

### Bootstrapped DEA – Coercive measures

For the percentage of cases treated without coercion, the mean TE score was 0.91 (*SD* = 0.03; range = 0.85–0.96). This indicates that on average, a clinic’s percentage of cases treated without coercion could be improved by 9% while keeping inputs constant. Clinic 6 had the highest TE score with 0.96 (lower CI = 0.94, upper CI = 0.99). With 23.54 FTE per 100 beds for nurses, 78.33 FTE per 100 beds for physicians, and 32.34 FTE per 100 beds for other staff, this clinic treated 98.6% of its 855 cases without coercion. In total, it applied 12 coercive measures (see Table 1). Clinic 19 had the lowest TE score with 0.85 (lower CI = 0.84, upper CI = 0.87). Employing 41.45 FTE per 100 beds for nurses, 89.37 FTE per 100 beds for physicians, and 57.49 FTE per 100 beds for other staff, this clinic treated 86.42% of its 1,325 cases without coercion. In total, it applied 180 coercive measures (see Table 1).

Bootstrapped TE scores with confidence intervals of each clinic are reported in Table 2.

**Table 2.** Bootstrapped DEA results for cases treated without coercion

Clinic number	VRS TE	CI lower (VRS TE)	CI upper (VRS TE)
1	0.91	0.9	0.93
2	0.92	0.9	0.94
3	0.88	0.87	0.89
4	0.89	0.87	0.93
5	0.95	0.92	0.99
6	0.96	0.94	0.99
7	0.89	0.88	0.9
8	0.94	0.94	0.96
9	0.89	0.88	0.91
10	0.86	0.85	0.88
11	0.89	0.88	0.9
12	0.9	0.89	0.91
13	0.88	0.86	0.9
14	0.92	0.9	0.94
15	0.93	0.9	0.99
16	0.93	0.87	1.01
17	0.94	0.9	1
18	0.94	0.94	0.95
19	0.85	0.84	0.87
20	0.91	0.91	0.92
21	0.89	0.88	0.91
22	0.94	0.92	0.96
Mean	0.91	0.89	0.94
SD	0.03	0.03	0.04
Median	0.91	0.90	0.93
Range	0.85–0.96	0.84–0.94	0.87–1.01

Abbreviation: VRS TE, technical efficiency scores computed with variable returns to scale.

Bootstrapped DEA results for specific coercion types are reported in Supplementary Tables S1–S4. Mean TE scores were 0.75 (*SD* = 0.08; range = 0.65–0.91) for the reversed cumulative duration of seclusions, 0.79 (*SD* = 0.12; range = 0.52–0.95) for the reversed cumulative duration of fixations, 0.79 (*SD =* 0.05; range = 0.68–0.91) for reversed coercive medications per case; 0.66 (*SD =* 0.19; range = 0.21–0.88) for reversed movement restrictions to bed or chair per case.

### Bootstrapped DEA – HoNOS

For the difference in HoNOS scores between the beginning and end of inpatient treatment, the mean TE score was 0.66 (*SD* = 0.11, range = 0.50–0.87). This indicates that on average, a clinic’s difference in HoNOS scores could be improved by 34% while keeping inputs constant. Clinic 19 had the highest TE score with 0.87 (lower CI = 0.85, upper CI = 0.92). With 41.45 FTE per 100 beds for nurses, 89.37 FTE per 100 beds for physicians, and 57.49 FTE per 100 beds for other staff, this clinic reached an average HoNOS difference of 6.33. Clinic 20 had the lowest TE score with 0.5 (lower CI = 0.47, upper CI = 0.54). Employing 45.83 FTE per 100 beds for nurses, 155.7 FTE per 100 beds for physicians, and 52.63 FTE per 100 beds for other staff, this clinic reached an average HoNOS difference of 1.74.

Bootstrapped TE scores with confidence intervals of each clinic are reported in Table 3.

**Table 3.** Bootstrapped DEA results for HoNOS difference

Clinic number	VRS TE	CI lower (VRS TE)	CI upper (VRS TE)
1	0.56	0.55	0.6
2	0.86	0.81	0.93
3	0.55	0.54	0.59
4	0.67	0.61	0.78
5	0.64	0.57	0.76
6	0.59	0.55	0.68
7	0.5	0.49	0.53
8	0.7	0.68	0.75
9	0.81	0.78	0.86
10	0.68	0.66	0.73
11	0.6	0.58	0.64
12	0.61	0.59	0.65
13	0.8	0.76	0.88
14	0.65	0.61	0.72
15	0.72	0.59	0.96
16	0.65	0.4	1.09
17	0.72	0.59	0.97
18	NA	NA	NA
19	0.87	0.85	0.92
20	0.5	0.47	0.54
21	0.63	0.61	0.68
22	0.64	0.61	0.69
Mean	0.66	0.61	0.76
SD	0.11	0.11	0.15
Median	0.65	0.59	0.73
Range	0.50–0.87	0.40–0.85	0.53–1.09

Abbreviation*:* VRS TE, technical efficiency scores computed with variable returns to scale.

### BSCL

For the difference in BSCL scores between the beginning and end of inpatient treatment, the mean TE score was 0.82 (*SD* = 0.08; range = 0.69–0.95). This indicates that on average, a clinic’s difference in BSCL scores could be improved by 18% while keeping inputs constant. Clinic 19 had the highest TE score with 0.95 (lower CI = 92, upper CI = 1). Treating 1,325 cases with 41.45 FTE per 100 beds for nurses, 89.37 FTE per 100 beds for physicians, and 57.49 FTE per 100 beds for other staff, this clinic reached an average BSCL difference of 37.53. Clinic 15 had the lowest TE score with 0.69 (lower CI = 0.63, upper CI = 0.83). Treating 1,906 cases with 17.6 FTE per 100 beds for nurses, 107.23 FTE per 100 beds for physicians, and 9.24 FTE per 100 beds for other staff, this clinic reached an average BSCL difference of 26.67.

Bootstrapped TE scores with confidence intervals of each clinic are reported in Table 4.

**Table 4.** Bootstrapped DEA results for BSCL difference

Clinic number	VRS TE	CI lower (VRS TE)	CI upper (VRS TE)
1	0.7	0.68	0.72
2	0.85	0.82	0.89
3	0.8	0.78	0.83
4	0.87	0.83	0.95
5	0.73	0.68	0.8
6	0.77	0.74	0.83
7	0.87	0.85	0.91
8	0.76	0.74	0.78
9	0.92	0.85	0.97
10	0.88	0.86	0.92
11	0.88	0.86	0.92
12	0.84	0.81	0.88
13	0.9	0.87	0.95
14	0.94	0.9	1
15	0.69	0.63	0.83
16	0.84	0.69	1.05
17	0.84	0.76	0.99
18	NA	NA	NA
19	0.95	0.92	1
20	0.74	0.71	0.77
21	0.73	0.71	0.76
Mean	0.82	0.78	0.89
SD	0.08	0.08	0.09
Median	0.84	0.80	0.9
Range	0.69–0.95	0.63–0.92	0.72–1.05

Abbreviation: VRS TE, technical efficiency scores computed with variable returns to scale.

### Truncated regression

Visual inspection of scatterplots provided no clear hints for associations between clinic size and TE scores of the investigated outcome measures (Supplementary Figures S1–S7). Truncated regression analyses revealed no significant effect of clinic size on TE scores regarding any of the investigated outcome measures (Table 5).

**Table 5.** Truncated regression results

	Estimate	SE	*t*-statistic	*p*-value
VRS TE (cases treated without coercion) ~ inpatient cases				
Intercept	1.09	0.02	66.81	<.001
Inpatient cases	0.000005	0.000007	0.79	.427
VRS TE (reversed cumulative duration of isolations) ~ inpatient cases				
Intercept	1.39	0.08	18.22	<.001
Inpatient cases	−0.00002	0.00003	−0.71	.477
VRS TE (reversed cumulative duration of fixations) ~ inpatient cases				
Intercept	0.92	0.75	1.21	.225
Inpatient cases	−0.00006	0.0002	−0.34	.73
VRS TE (reversed coercive medications per case) ~ inpatient cases				
Intercept	1.22	0.04	28.61	<.001
Inpatient cases	0.00003	0.00002	1.43	.15
VRS TE (reversed movement restrictions per case) ~ inpatient cases				
Intercept	−10.02	30.09	−0.33	.74
Inpatient cases	0.002	0.006	0.25	.799
VRS TE (HoNOS difference) ~ inpatient cases				
Intercept	1.39	0.01	11.06	<.001
Inpatient cases	0.00007	0.00006	1.29	.198
VRS TE (BSCL difference) ~ inpatient cases				
Intercept	1.12	0.07	16.6	<.001
Inpatient cases	0.00005	0.00003	1.81	.069

Abbreviation: VRS TE, technical efficiency scores computed with variable returns to scale.

## Discussion

We compared Swiss psychiatric clinics regarding their relative efficiency in treating cases without coercion given their staff resources. As a secondary aim, we compared the clinics’ relative efficiencies for self-reports and third-person reports of changes of symptom severity during inpatient stay. For all outcome measures, our results suggest that clinics’ efficiencies may be influenced by management factors independent of staff resources or clinic size. The current study shows that DEA may be helpful to guide public decision-making regarding the efficient reduction of coercion or symptom severity.

Preliminary analyses revealed a strong positive correlation between the percentage of cases treated with the total coercion numbers. Similar correlations between absolute and relative measures could be observed for specific coercion types. Thus, it can be assumed that the results of our analyses focusing on relative measures of coercion also entail information about their absolute numbers.

Our results suggest that on average, the included clinics could improve their percentage of cases treated without coercion by 9% via organizational changes through management while keeping staff numbers constant. In a sample of 12 German clinics with the same input and output variables, this percentage was 2.9% at maximum over three investigated years [27]. While efficiency scores are always computed in relation to the current sample and thus cannot be directly compared between studies, we regard this as a sign of greater variation and thus potential for change regarding in the present sample. This is supported by the fact that the number of cases treated without coercion was lower in the present sample (Mean = 92.21; *SD* = 3.39) than in the compared study (Mean = 95.58%; *SD* = 2.47%).

Our additional analyses of specific coercive measures suggest that there is potential for efficiency improvements across all analyzed types of coercion. This potential seems to be especially high for movement restrictions to a bed or chair. A reason for this may be that half of the included clinics kept these restrictions at a minimum of zero to three instances in total. These numbers illustrate the potential for a reduction of movement restrictions.

We found no evidence of an association between clinic size and the clinics’coercion reduction efficiency. This result is in line with previous studies finding either weak or no associations of clinic-level characteristics such as size or staff to bed ratio with coercion [18, 20].Comparable to our findings, both studies report unexplained variance in the use of coercion between clinics. The effectiveness of previous coercion prevention programs suggest that efficient coercion reduction is managementrelated.An important recent development in this regard are the recommendations to prevent coercion by the World Psychiatry Association [50]. Potential for improvement may occur on different organizational levels. On the staff level, de-escalation trainings are associated with a reduction of coercion [29]. On the ward level, changes in size, architecture, and opportunities for meaningful activities and social interaction facilitate a preventive environment [51]. As a recent example, architectural changes to psychiatric wards of the Vienna General Hospital were accompanied by decreased cumulative durations of coercive measures [52].Interventions like the Six Core Strategies to Reduce Seclusion and Restraint [53], and open-door policies [19, 28] combine changes of hospital structure, management style, and staff trainings for a reduction of coercive measures. For example, the open doors program at the Universitary Psychiatric clinics in Basel has resulted in a continuous, long-term reduction of coercive measures [19, 54]. Extending the scope of coercion prevention beyond inpatient treatment, the community treatment program ACCESS model for patients with schizophrenia spectrum disorders poses a way to reduce involuntary admissions [55].

Secondary outcomes suggest that clinics could improve changes of third-person reports of symptom severity by 34% and changes of self-reported symptom severity by 18% while keeping staff numbers constant. A reason for this difference may be that third-person symptom ratings reflect staff-dependent variability in the perception and classification of patients between clinics. Further, it needs to be noted that the two symptom ratings differed in response rates (see Methods Section). We found no effect of clinic size on efficiency scores regarding changes of self-reports or third-person reports of symptom severity. Further, it can be assumed that efficiency scores were independent of the distribution of psychiatric disorders among the clinics, as both symptom measures were adjusted for patient mix prior to our analyses. It remains to be investigated which other clinic-level factors may influence a clinic’s efficiency regarding symptom reduction. Investigations of multicenter routine data suggest that the socioeconomic mix of patients (an aspect of so-called neighborhood effects) and the type of clinic (primary care, secondary care or universitary hospital) may influence treatment outcomes [56, 57]. Importantly, both of these factors are the result of unpreventable between-clinic variability. It therefore remains unclear how much efficiency scores regarding changes of symptom severity can be influenced by management factors, such as staff trainings or organizational changes.

### Limitations

The following limitations should be considered regarding the results of the current study. As described in the Methods section, several clinics had to be excluded due to issues in data reporting. For the same reasons, we cannot evaluate whether exclusions affected the results systematically. However, about 60% of all psychiatric hospitals working with coercive measures in acute psychiatric inpatient treatment in Switzerland across all language regions were included in the current study, increasing generalizability of our findings. Due to the limited sample size, only one predictor was included in the truncated regression models.

Regarding the analysis, it is important to note that technical efficiency scores are always computed relative to the given sample and thus cannot be directly compared across studies. The bootstrap procedure encounters the problem of overestimation of TE scores [44] in a statistical manner. Yet, we want to caution the reader to regard TE scores as a broad orientation for the potential for change rather than as a precise estimate of how much a given set of clinics could or should improve with regard to a specific outcome measure.

As mentioned above, neighborhood effects may have contributed to between-clinic variability that cannot be influenced by management. We assume such effects to be stronger for symptom change scores than for coercive measures. Symptom reduction reflects a treatment process patients contribute to over days or weeks. In this time frame, many opportunities for utilization of personal financial or educational resources occur. In contrast, coercive measures are the result of single, situational decisions made by the staff. Yet, we could not test this assumption empirically.

This study is based on the assumption that a further reduction of coercive measures in Swiss psychiatric clinics is possible. Yet, coercive measures may at times be necessary to ensure safety, especially for patients entering treatment with high levels of aggression. In addition, some patients retrospectively regard coercive measures as a necessary part of treatment [2].

The current study is bound to objective measures of the quantity of coercive measures. Yet, carefully applied coercive measures can be associated with less feelings of coercion and better treatment outcomes than seemingly more voluntary treatment options [58]. These aspects of coercion are not captured by the clinical routine documentation of coercive measures that our study relied on.

### Recommendations for future research

To our knowledge, this is the first study to investigate the efficiency of psychiatric clinics regarding coercion reduction and symptom change in Switzerland. Our results show that DEA has the potential to differentiate between clinics regarding their capacity to efficiently reduce coercion and symptom severity independent of staff resources. It therefore may be fit to guide public health decision making in these regards.

While our results differentiated between clinics regarding efficiency scores, it remains an open question how much the observed differences between clinics change or remain stable over time. Longitudinal DEA investigations could reveal important information in this regard. For example, clinics consistently showing high levels of efficiency regarding would be especially fit for closer investigation to derive best-practice recommendations.

By now, a multitude of recommendations for coercion reduction via interventions on the organizational level exist. For future studies, it would be interesting to quantify how much these changes have been implemented on a clinic level. In combination with DEA, such measures would allow for an investigation of which organizational changes have the strongest effect on clinics´ efficiency regarding coercion reduction or symptom change.

Other than for coercion reduction, we know of no recommendations on how to structure a clinic for efficient treatment of psychiatric symptoms. A reason for this may be that many partial aspects of treatment (medication, psychotherapeutic techniques, etc.) are tested in this regard prior to their application as standard treatment. Yet, during inpatient treatment these methods are applied in a larger clinical context with a distinct organizational culture [59]. Thus, an aim for future research could be the identification organizational changes that have the potential to influence efficient symptom reduction.

## Conclusion

We compared Swiss psychiatric clinics regarding their relative efficiency in treating cases without coercion given their staff resources. As a secondary aim, we compared the clinics’ relative efficiencies for self-reports and third-person reports of changes of symptom severity during inpatient stay. For all outcome measures, our results suggest that clinics’ efficiencies may be influenced by management factors independent of staff resources or the clinic’s size. Among specific types of coercion, the potential for efficiency improvements via management was highest for fixations and other movement restrictions. Our results underline the importance of management factors for efficient reduction of coercion and psychiatric symptoms during inpatient stays. In future research, longitudinal DEA may be applied to investigate which interventions have the potential to efficiently reduce coercive measures and influence other psychiatric treatment outcomes.

## Supporting information

10.1192/j.eurpsy.2025.10034.sm001 Müller et al. supplementary material Müller et al. supplementary material

## Data Availability

We combined publicly available data on coercive measures and symptom severity from the Swiss National Association for Quality Development (www.anq.ch/en/) in Hospitals and Clinics and data on staff numbers from the Federal Office of Public Health (https://bag.admin.ch/bag/en/home.html).

## Supplementary material

The supplementary material for this article can be found at http://doi.org/10.1192/j.eurpsy.2025.10034.
