# Supplementary material for: Relative efficiency of psychiatric clinics in treating cases without coercion and achieving symptom reduction
Source: Eur Psychiatry. 2025 May 27;68(1):e76. doi: 10.1192/j.eurpsy.2025.10034 (PMC12174862; doi:10.1192/j.eurpsy.2025.10034)

| Clinic  Number | VRS TE | CI lower  (VRS TE) | CI upper  (VRS TE) |
| --- | --- | --- | --- |
| 1 | 0.67 | 0.64 | 0.72 |
| 2 | 0.68 | 0.63 | 0.76 |
| 3 | 0.66 | 0.64 | 0.71 |
| 4 | NA | NA | NA |
| 5 | NA | NA | NA |
| 6 | 0.65 | 0.58 | 0.74 |
| 7 | 0.85 | 0.83 | 0.91 |
| 8 | 0.83 | 0.8 | 0.89 |
| 9 | 0.71 | 0.68 | 0.78 |
| 10 | 0.81 | 0.77 | 0.88 |
| 11 | 0.75 | 0.72 | 0.82 |
| 12 | 0.69 | 0.66 | 0.74 |
| 13 | 0.66 | 0.6 | 0.74 |
| 14 | 0.75 | 0.68 | 0.83 |
| 15 | 0.76 | 0.55 | 0.98 |
| 16 | 0.76 | 0.55 | 1 |
| 17 | NA | NA | NA |
| 18 | NA | NA | NA |
| 19 | 0.83 | 0.77 | 0.92 |
| 20 | 0.87 | 0.85 | 0.93 |
| 21 | 0.67 | 0.62 | 0.73 |
| 22 | 0.91 | 0.84 | 1 |
| Mean | 0.75 | 0.69 | 0.84 |
| SD | 0.08 | 0.1 | 0.1 |
| Median | 0.75 | 0.67 | 0.82 |
| Range | 0.65 – 0.91 | 0.55 – 0.85 | 0.71 – 1 |

# Supplementary Tables

Supplementary Table 1. Bootstrapped DEA Results for Reversed Cumulative Duration of Seclusions

*Note.* The cumulative duration of seclusions was computed by frequency x time (in hours) of seclusions on the case level. NA = not available (clinic excluded due to outlier values).

| Clinic  Number | VRS TE | CI lower  (VRS TE) | CI upper  (VRS TE) |
| --- | --- | --- | --- |
| 1 | 0.79 | 0.77 | 0.83 |
| 2 | 0.56 | 0.53 | 0.61 |
| 3 | 0.82 | 0.81 | 0.85 |
| 4 | 0.68 | 0.62 | 0.78 |
| 5 | 0.52 | 0.47 | 0.59 |
| 6 | 0.84 | 0.73 | 0.97 |
| 7 | 0.88 | 0.86 | 0.91 |
| 8 | 0.94 | 0.92 | 0.98 |
| 9 | 0.85 | 0.82 | 0.89 |
| 10 | 0.94 | 0.91 | 0.98 |
| 11 | 0.88 | 0.86 | 0.93 |
| 12 | 0.83 | 0.82 | 0.86 |
| 13 | 0.84 | 0.79 | 0.91 |
| 14 | 0.58 | 0.54 | 0.64 |
| 15 | 0.78 | 0.6 | 1.13 |
| 16 | 0.78 | 0.61 | 1.21 |
| 17 | NA | NA | NA |
| 18 | 0.95 | 0.93 | 0.98 |
| 19 | 0.84 | 0.79 | 0.91 |
| 20 | 0.8 | 0.79 | 0.82 |
| 21 | 0.66 | 0.64 | 0.7 |
| 22 | 0.9 | 0.85 | 0.98 |
| Mean | 0.79 | 0.75 | 0.88 |
| SD | 0.12 | 0.14 | 0.16 |
| Median | 0.83 | 0.79 | 0.91 |
| Range | 0.52 – 0.95 | 0.47 – 0.93 | 0.59 – 1.21 |

Supplementary Table 2. Bootstrapped DEA Results for Reversed Cumulative Duration of Fixations

*Note.* The cumulative duration of fixations was computed by frequency x time (in hours) of fixations on the case level. NA = not available (clinic excluded due to outlier values).

Supplementary Table 3. Bootstrapped DEA Results for Reversed Coercive Medications per Case

| Clinic  Number | VRS TE | CI lower  (VRS TE) | CI upper  (VRS TE) |
| --- | --- | --- | --- |
| 1 | 0.75 | 0.73 | 0.78 |
| 2 | 0.73 | 0.69 | 0.78 |
| 3 | NA | NA | NA |
| 4 | 0.76 | 0.7 | 0.84 |
| 5 | 0.84 | 0.77 | 0.94 |
| 6 | 0.8 | 0.73 | 0.9 |
| 7 | 0.73 | 0.72 | 0.77 |
| 8 | 0.79 | 0.77 | 0.83 |
| 9 | 0.75 | 0.73 | 0.79 |
| 10 | 0.83 | 0.81 | 0.88 |
| 11 | 0.83 | 0.81 | 0.88 |
| 12 | 0.81 | 0.8 | 0.85 |
| 13 | 0.77 | 0.73 | 0.82 |
| 14 | 0.91 | 0.85 | 1 |
| 15 | 0.83 | 0.7 | 0.98 |
| 16 | 0.81 | 0.67 | 1.01 |
| 17 | 0.82 | 0.67 | 1.01 |
| 18 | 0.79 | 0.77 | 0.82 |
| 19 | 0.71 | 0.67 | 0.76 |
| 20 | NA | NA | NA |
| 21 | 0.68 | 0.66 | 0.73 |
| 22 | 0.77 | 0.73 | 0.82 |
| Mean | 0.79 | 0.74 | 0.86 |
| SD | 0.05 | 0.05 | 0.09 |
| Median | 0.79 | 0.73 | 0.84 |
| Range | 0.68 – 0.91 | 0.66 – 0.85 | 0.73 – 1.01 |

*Note.* NA = not available (clinic excluded due to outlier values).

| Clinic  Number | VRS TE | CI lower  (VRS TE) | CI upper  (VRS TE) |
| --- | --- | --- | --- |
| 1 | 0.39 | 0.37 | 0.42 |
| 2 | 0.83 | 0.75 | 0.95 |
| 3 | 0.85 | 0.82 | 0.93 |
| 4 | 0.57 | 0.45 | 0.74 |
| 5 | 0.74 | 0.54 | 0.99 |
| 6 | 0.79 | 0.63 | 1.01 |
| 7 | 0.64 | 0.62 | 0.7 |
| 8 | 0.46 | 0.44 | 0.5 |
| 9 | 0.59 | 0.56 | 0.66 |
| 10 | 0.86 | 0.81 | 0.95 |
| 11 | 0.63 | 0.59 | 0.69 |
| 12 | 0.87 | 0.84 | 0.95 |
| 13 | 0.49 | 0.44 | 0.57 |
| 14 | 0.85 | 0.75 | 1.01 |
| 15 | 0.54 | 0.13 | 1.31 |
| 16 | 0.43 | -0.09 | 2.77 |
| 17 | 0.58 | 0.22 | 1.18 |
| 18 | 0.88 | 0.85 | 0.96 |
| 19 | 0.21 | 0.19 | 0.25 |
| 20 | 0.59 | 0.57 | 0.64 |
| 21 | 0.84 | 0.78 | 0.94 |
| 22 | 0.86 | 0.78 | 0.99 |
| Mean | 0.66 | 0.55 | 0.91 |
| SD | 0.19 | 0.26 | 0.49 |
| Median | 0.64 | 0.58 | 0.94 |
| Range | 0.21 –  0.88 | -0.09 – 0.85 | 0.25 – 2.77 |

Supplementary Table 4. Bootstrapped DEA Results for Reversed Movement Restrictions per Case

# Supplementary Figures

Supplementary Figure 1. VRS TE Scores for the Percentage of Cases Treated without Coercion in Relation to the Total Case Number


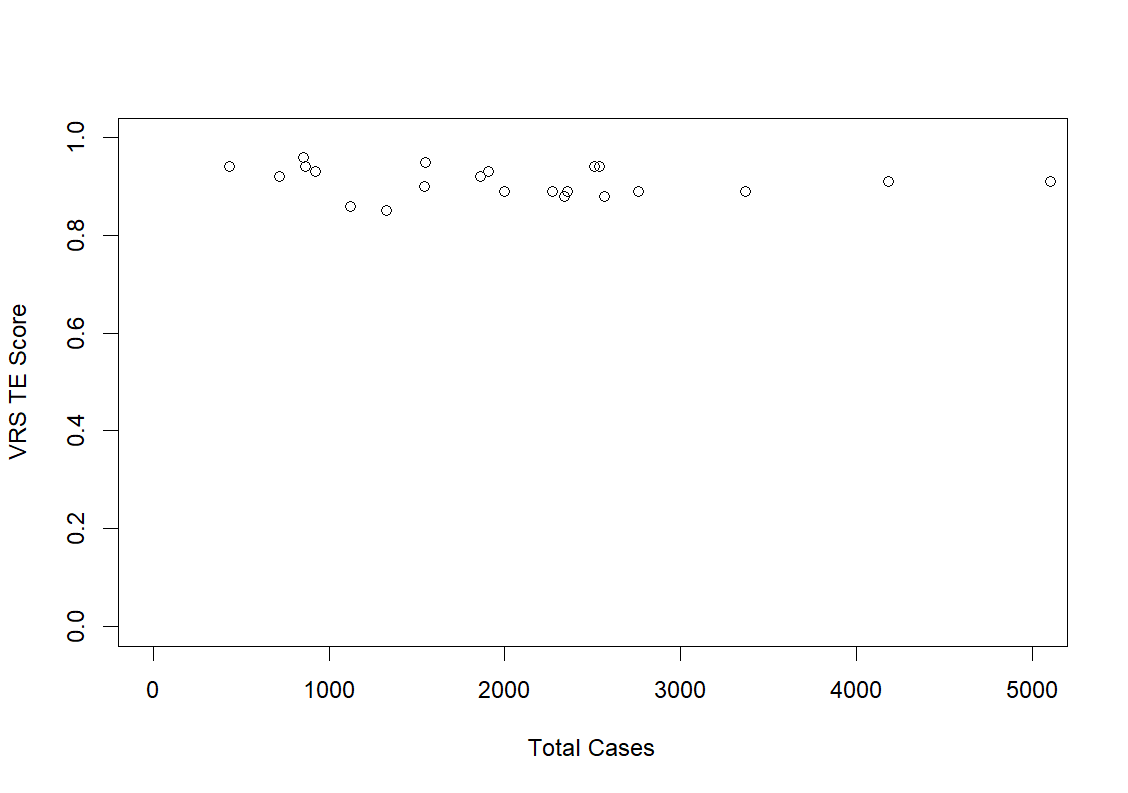


Supplementary Figure 2. VRS TE Scores for the HoNOS Difference in Relation to the Total Case Number


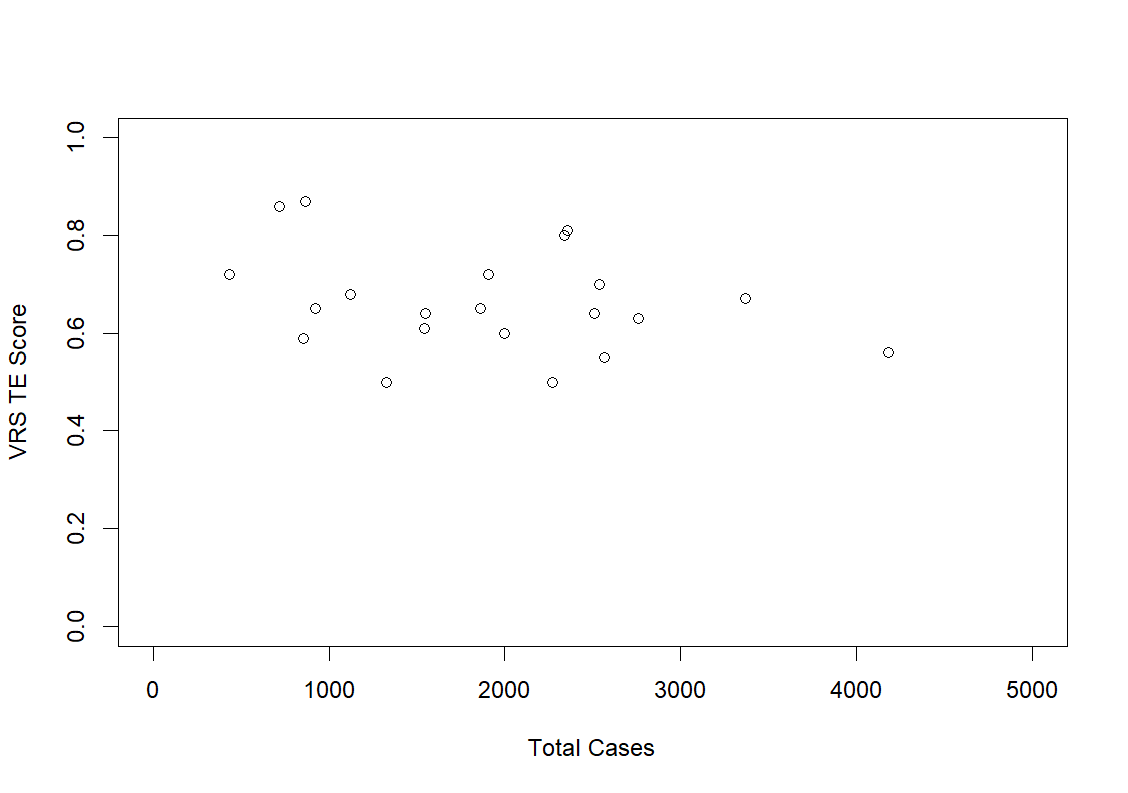


Supplementary Figure 3. VRS TE Scores for the BSCL Difference in Relation to the Total Case Number


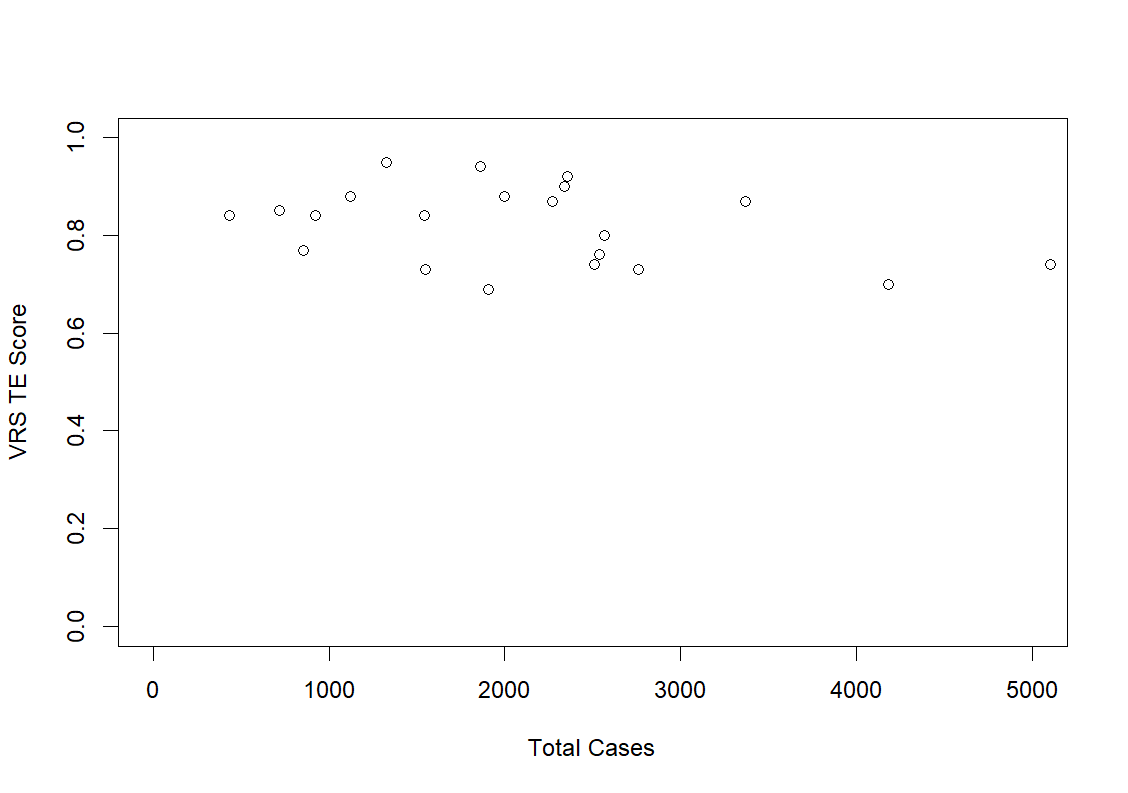


Supplementary Figure 4. VRS TE Scores for the Reversed Cumulative Duration of Seclusions in Relation to the Total Case Number


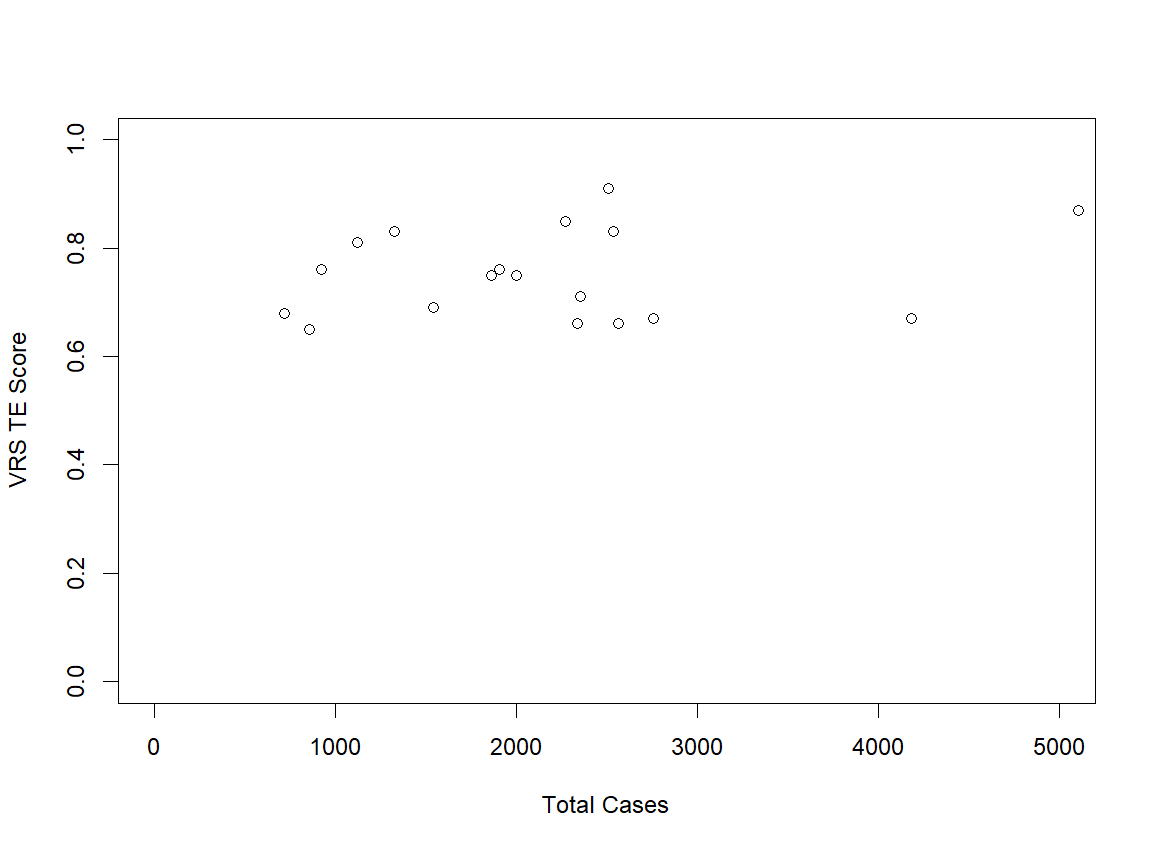


Supplementary Figure 5. VRS TE Scores for the Reversed Cumulative Duration of Fixations in Relation to the Total Case Number


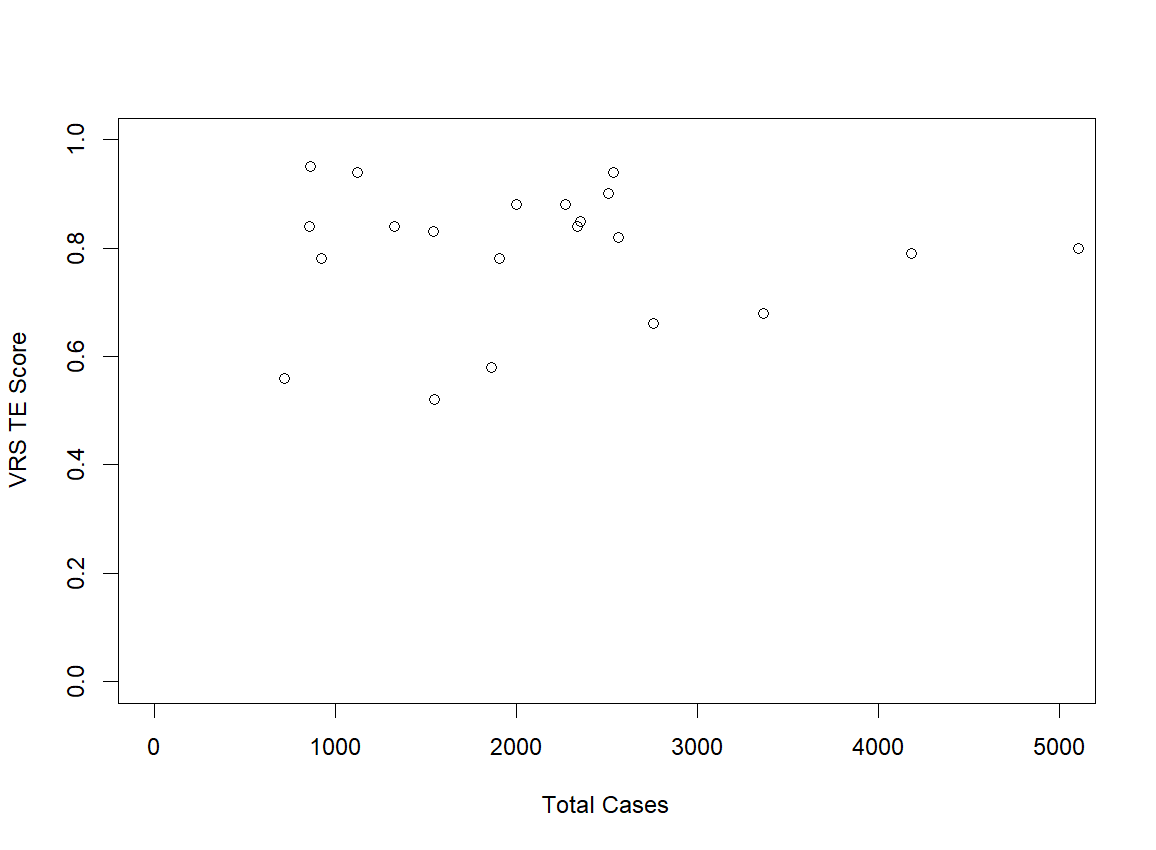


Supplementary Figure 6. VRS TE Scores for the Reversed Number of Coercive Medications per Case in Relation to the Total Case Number


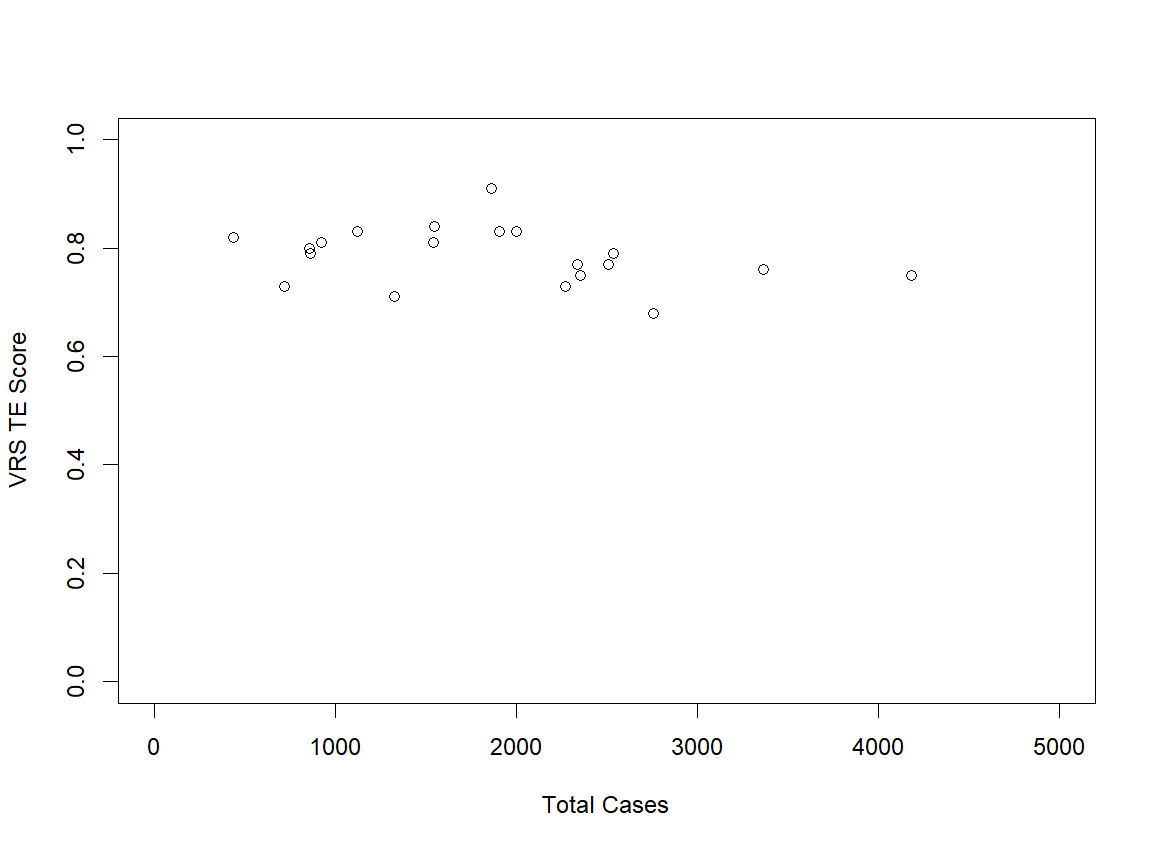


Supplementary Figure 7. VRS TE Scores for the Reversed Number of Movement Restrictions per Case in Relation to the Total Case Number


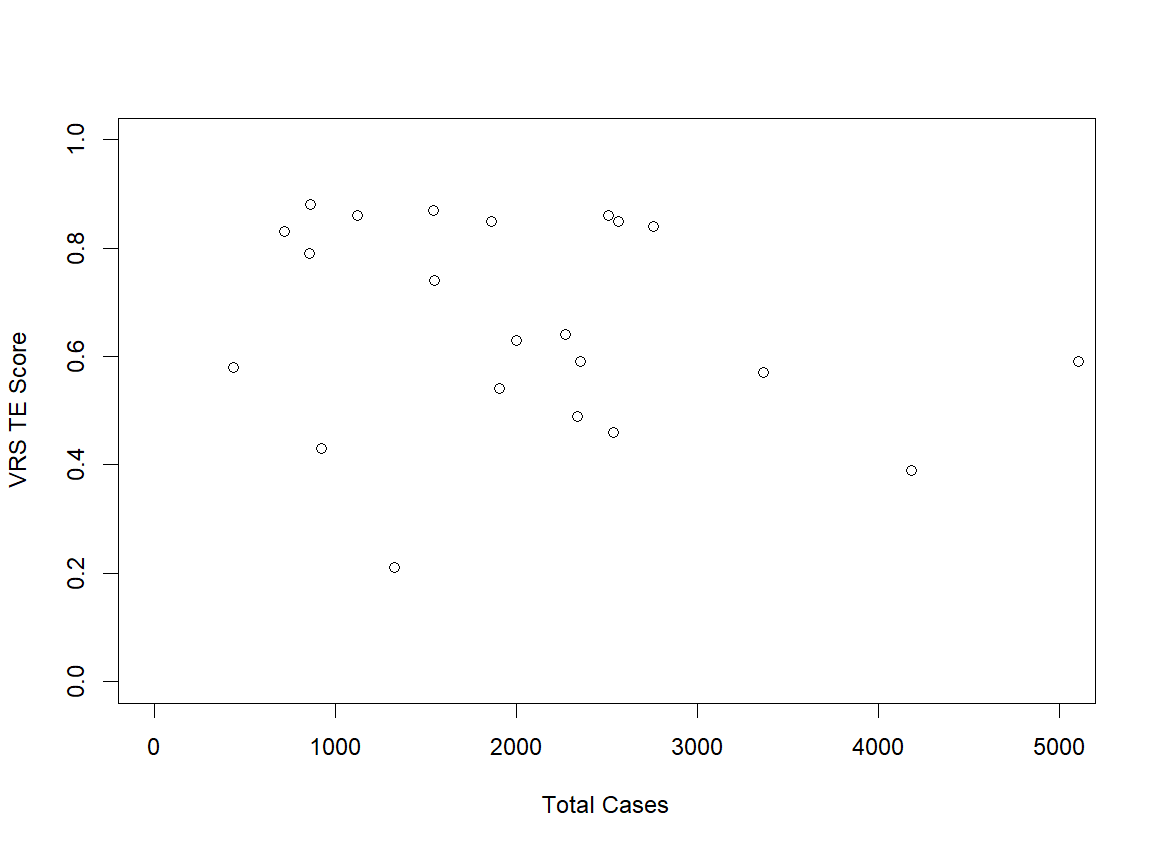

Supplement: Müller et al. supplementary material [file S0924933825100345sup001.docx]
